# Biological activities of extracts from *Chenopodium ambrosioides* Lineu and *Kielmeyera neglecta* Saddi

**DOI:** 10.1186/1476-0711-11-20

**Published:** 2012-07-28

**Authors:** Zulane Lima Sousa, Fernando Faustino de Oliveira, Aline Oliveira da Conceição, Luiz Alberto Mattos Silva, Maria Helena Rossi, Juliana da Silva Santos, João Luciano Andrioli

**Affiliations:** 1 Programa de Pós-graduação em Biologia e Biotecnologia de Microrganismos, Universidade Estadual de Santa Cruz, Campus Prof. Soane Nazaré de Andrade, Km 16 – Rodovia Ilhéus/Itabuna, 45662-900, Ilhéus, Brazil; 2 Departamento de Ciências Exatas e Tecnológicas, Universidade Estadual de Santa Cruz, Campus Prof. Soane Nazaré de Andrade, Km 16 – Rodovia Ilhéus/Itabuna, 45662-900, Ilhéus, Brazil; 3 Departamento de Ciências Biológicas, Universidade Estadual de Santa Cruz, Campus Prof. Soane Nazaré de Andrade, Km 16 – Rodovia Ilhéus/Itabuna, 45662-900, Ilhéus, Brazil; 4 Instituto Biológico, Centro de Sanidade Animal, Av. Cons. Rodrigues Alves, 1252, 04014-002, São Paulo, Brazil

**Keywords:** Antimicrobial, Bioactivity, Extract, *Chenopodium ambrosioides*, *Kielmeyera neglecta*, *Candida*

## Abstract

**Background:**

*Chenopodium ambrosioides and Kielmeyera neglecta* are plants traditionally used in Brazil to treat various infectious diseases. The study of the biological activities of these plants is of great importance for the detection of biologically active compounds.

**Methods:**

Extracts from these plants were extracted with hexane (Hex), dichloromethane (DCM), ethyl acetate (EtOAc) and ethanol (EtOH) and assessed for their antimicrobial properties, bioactivity against *Artemia salina* Leach and antifungal action on the cell wall of *Neurospora crassa*.

**Results:**

Extracts from *C. ambrosioides* (Hex, DCM and EtOH) and *K. neglecta* (EtOAc and EtOH) showed high bioactivity against *A. salina* (LD50 < 1000 μg/mL), which might be associated with cytotoxic activity against cancer cells. *C. ambrosioides* Hex and DCM showed specific activity against yeasts, highlighting the activity of hexanic extract against *Candida krusei* (MIC = 100 μg/mL). By comparing the inhibitory concentration of 50% growth (IC 50%) with the growth control, extracts from *K. neglecta* EtOAc and EtOH have shown activities against multidrug-resistant bacteria (*Enterococcus faecalis* ATCC 51299 and *Staphylococcus aureus* ATCC 43300), with IC 50% of 12.5 μg/mL The assay carried out on *N. crassa* allowed defining that extracts with antifungal activity do not have action through inhibition of cell wall synthesis.

**Conclusions:**

Generally speaking, extracts from *C. ambrosioides* and *K. neglecta* showed biological activities that have made the search for bioactive substances in these plants more attractive, illustrating the success of their use in the Brazilian folk medicine.

## Background

The observed medical advances over the last years has led to an increase in the survival of immunocompromised people; because this population group is affected by some disease that suppresses the immune system, such as AIDS, or due to intensive use of chemotherapy or immunosuppressive drugs, these people are more susceptible to opportunistic infections like candidiasis, which are considered difficult to treat in these cases [1-3]. In addition to this, there is an increase in the number of microorganisms resistant or multiresistant to antibiotics [3-5], such as the methicillin-resistant *Staphylococcus aureus* (MRSA), which is one of the major bacterial species causing nosocomial infections worldwide [6]. Together, these factors stimulate the search for new drugs that are effective and less toxic to patients [1,2,7]. In this context, natural products have been historically and continue to be the focus of researches on antimicrobial drugs [8], the main source of which is to be found in plants [9].

The use of plants in the treatment of infectious diseases is common since ancient times [10], evidencing its great potential in the research of antimicrobial components. This potential can be explained by the large variety and complexity of secondary metabolites synthesized by plants as a result of adaptive and defensive mechanisms against insects, animals and microorganisms [11]. Hence, plants are regarded as a great laboratory of organic synthesis, as a result of millions of years of adaptation, providing an invaluable source of new molecules for researching antimicrobial activities [12-14].

It is of great interest that experimental studies of new drugs are conducted using ethnopharmacology, i.e., the study of biological activities of plants with medicinal use supported by popular knowledge [15], since this approach has a great potential to find new bioactive substances [16].

*Chenopodium ambrosioides* Lineu is an herb of the Chenopodiaceae family, indigenous to South America, with broad geographic distribution in Brazil, popularly known as “mastruz” [17-19]; furthermore, this plant is traditionally and widely used as anti-parasitic, anti-inflammatory and antibiotic, which efficacy has been scientifically proven [18,20,21]. The essential oil of *C. ambrosioides* is known to inhibit the growth of yeast species [22], dermatophytes [23] and other filamentous fungi [23,24] and the hexane extract of *C. ambrosioides* is known to inhibit the growth of filamentous fungi [17]. However, further studies are needed with different microorganisms.

*Kielmeyera neglecta* Saddi is a plant of the *Clusiaceae* family, endemical to Southern Bahia, Brazil, and found in the Atlantic Forest [25]; this plant is popularly known as “pau-santo” (holy-wood) [26], term also used to denominate plants of the same genus [27-29], which are used in the Brazilian folk medicine to treat various infectious diseases [4]. Previous studies have demonstrated the existence of antibacterial and antifungal activities of extracts from these plants [2,30], but additional studies are still needed.

Apart from dealing with the assessment of different extracts from *C. ambrosioides* and *K. neglecta* as for their antibacterial and antifungal properties, this study addresses the bioactivity against *Artemia salina* Leach and the antifungal effect on the cell wall of *Neurospora crassa*.

## Methods

### Plant material

Branches containing leaves and seeds of *C. ambrosioides* were collected in Ilhéus, whereas leaves from *K. neglecta* were collected in Una, both cities in the state of Bahia, Brazil. Data collection was performed in May 2010; the material was identified by the teacher Luiz Alberto Silva Mattos and voucher specimens were deposited at the herbarium of Universidade Estadual de Santa Cruz (HUESC).

### Preparation of extracts

After drying in a ventilated oven at 50°C, for 5 h, aerial parts of *C. ambrosioides* (633.3 g) and leaves of *K. neglecta* (1783.0 g) were ground and extracted by exhaustive maceration in sequence with the solvents hexane (Hex), dichloromethane (DCM), ethyl acetate (EtOAc) and ethanol (EtOH); after filtration through filter paper, solvents were removed by a rotary evaporator, under reduced pressure, and temperature below the boiling point of the solvent, thus obtaining extracts Hex (15.2 g), DCM (18.6 g), EtOAc (9.2 g) and EtOH (34.2 g) for *C. ambrosioides* and Hex (28.3 g), DCM (47.7 g), EtOAc (16.4 g) and EtOH (35.0 g) for *K. neglecta*. All extracts were kept in glass flasks, at room temperature, until their application. For the preparation of solutions to be used in the bioactivity against *Artemia salina*, all extracts were solubilized in ethanol so as to attain the desired concentration; regarding the other assays, EtOAc and EtOH extracts were resuspended in dimethyl sulfoxide, whereas Hex and DCM were resuspended in ethanol. The solutions for use in microbial assays were filtered through a 0.22 μm pore membrane and maintained refrigerated until use.

### Bacteria and fungi

The microorganisms used in the study were: *Enterococcus faecalis* ATCC 29212, *E. faecalis* ATCC 51299, *Escherichia coli* ATCC 25922, *E. coli* ATCC 35218, *Klebsiella pneumoniae* ATCC 700603, *Pseudomonas aeruginosa* ATCC 27853, *S. aureus* ATCC 29213, *S. aureus* ATCC 43300, *Candida albicans* ATCC 90028, *C. albicans* ATCC 10231, *C. parapsilosis* ATCC 90018, *C. parapsilosis* ATCC 22019, *C. krusei* ATCC 6258. The *N. crassa* isolate used to assess antifungal effects on the cell wall was kindly granted by Dr. Antonio Carlos Monteiro (UNESP, Jaboticabal, Brazil).

### Assay using *Artemia salina*

The assessment of bioactivity of extracts was carried out following the methodology described by Meyer et al. [31], with modifications. In order to obtain a nauplii population, crustacean cysts (INVE do Brasil, Fortaleza, Brazil) were incubated in sea water at room temperature, under direct light, for 48 h. The preparation of solutions with different concentrations of extract was performed by diluting the working solution in sea water with 1% Tween 80. These solutions were dispensed in 24-well plates to which ten nauplii have been added, and plates were incubated at room temperature, under direct light. After 6 and 24 h, the number of survivors was counted so as to determine the lethal concentration capable of eliminating 50% of organisms (LC_50_). The final concentrations of the tested extracts ranged between 100 and 2000 μg/mL; the concentration of Tween 80 was 0.94% and that of ethanol was below 1%. Negative and positive controls were simultaneously performed using the same concentrations of the working solvent and K_2_Cr_2_O_7_ (0.33 mM), respectively. The assays were performed in triplicate. LC_50_ was calculated using the probit method (BioStat, 2009), with a 95% confidence interval. The mortality was corrected using Abbott’s formula: ${\text{M}}_{\text{c}}=\left[{\text{M}}_{\text{t}}\left(\%\right)–{\text{M}}_{\text{nc}}\left(\%\right)/100–{\text{M}}_{\text{nc}}\left(\%\right)\right]\times 100$, where M_c_ = corrected mortality; M_t_ = mortality of the test; M_nc_ = mortality of negative control [32].

### Antimicrobial activity

Broth microdilution test was performed in accordance with the norms established by the Clinical and Laboratory Standards Institute (CLSI): M27-A6 [33], for bacteria, and M27-A2 [34], for yeasts.

Bacterial (10^8^ UFC/mL) and yeast cells (10^6^ UFC/mL) were inoculated in Mueller-Hinton broth and RPMI 1640 medium, respectively, in 96-well microdilution plates in the presence of extract at different concentrations, with final inoculum concentrations of 10^5^ UFC/mL for bacteria, and 10^3^ UFC/mL for yeasts. After 24 h of incubation at 35°C, the minimum inhibitory concentration (MIC) - considered the lowest concentration of the extract at which there was no visible growth of organisms - and the inhibitory concentration of 50% growth (IC 50%), defined as the lowest concentration of extract that can inhibit 50% of visible microbial growth have been determined. In cases of complete growth inhibition during the evaluation of antifungal activity, the minimum fungicidal concentration (MFC) defined as the lowest extract concentration that could completely eliminate the microorganism was confirmed by re-inoculation of 20 μL of the medium in microdilution culture plates in which there was no visible growth in Sabouraud Dextrose Agar, and incubated for 48 h at 35°C. The final concentrations of the tested extracts were 12.5-500 μg/mL for antibacterial activity, and 0.8-500 μg/mL for antifungal activity. Controls on growth, sterility of the medium, sterility of the extract, as well as negative (at the same concentrations of solvents) and positive controls (chloramphenicol at 50 μg/mL and amphotericin B at 5 μg/mL) were simultaneously conducted. All trials were performed in triplicate.

### Antifungal action on the cell wall of *N. crassa*

In the cell wall inhibition assay of *N. crassa*, the agar diffusion method allows macroscopic detection of inhibitors of fungal cell wall [35]. In appropriate medium and conditions and in the presence of cell wall inhibitors, the fungus grows as protoplasm, showing clear inhibition halo and undefined borders [36]. Evaluation of the antifungal action of extracts on the cell wall of *N. crassa* was performed following the methodology proposed by Boeck et al. [35]. *N. crassa* was grown on Sabouraud Dextrose Agar for 3 days, at room temperature, directly exposed to sunlight, thus producing an orange mycelium and spores; Using a Neubauer chamber, the spore inoculum was prepared and adjusted to 1 × 10^6^ spores/mL with a buffer solution containing 0.075 g/100 mL K_2_HPO_4,_ 0.10 g/100 mL KH_2_PO_4_ in a H_2_O 15:85 glycerol solution. Thirty milliliters of the osmotic medium with 0.5% peptone, 1.0% yeast extract, 4.0% sucrose and 1.5% agar has been autoclaved (121°C, 15 min). Shortly after, it was cooled to 45°C, 30 μL of the spore inoculum were added, and the solution was homogenized and placed in 90 mm diameter Petri plates; after solidification, 4.8 mm wells were made and 30 μL of solutions from the testing extract were dispensed at a concentration of 500 mg/mL achieved by diluting the working solution in distilled water. After incubation at room temperature, under direct light for 24 h, it has been macroscopically observed the presence of an inhibition halo. In the presence of cell wall inhibitors, the fungus grows as protoplasm, producing a halo of misty appearance. The solvents used for dilution of the extract have been used as a negative control, whereas ketoconazole (30 μg/mL) has been used as a positive control. The assay consisted of three separate trials.

## Results and discussion

**Figure 1:**
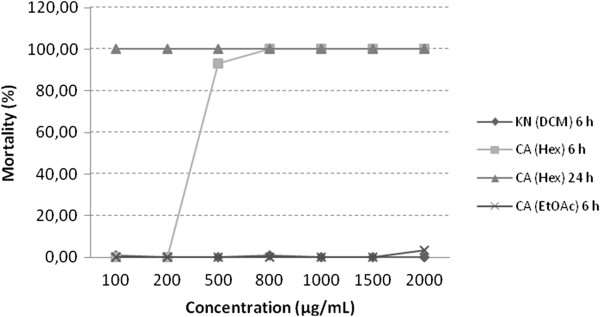
**Mortality of *Artemia salina *to extracts of *K. neglecta *(KN) and *C. ambrosioides *(CA); Hex, hexane; DCM, dichloromethane; EtOAc, ethyl acetate.**

The crustacean *A. salina* is highly sensitive to a variety of compounds [37] and is considered to be a useful screening tool of active substances in bioassay [4,31]; furthermore, it has good correlation with cytotoxicity in human cancer cells [31,38]. As shown in Table 1, results indicate high bioactivity (LC_50_ < 1000 μg/mL) for extracts from *K. neglecta* EtOAc and EtOH, and *C. ambrosioides* DCM and EtOH were considered as active [31,39,40]. As compared with the other tested extracts, *C. ambrosioides* Hex showed very high mortality rate (Figure 1) and thereby made it statistically impossible to calculate the LC_50_, but was considered to be an active extract. Bioactivity in 6 h was only high for *C. ambrosioides* Hex, reinforcing the possibility for this extract to contain bioactive compounds.

**Table 1:** **Bioactivity of extracts of *Chenopodium ambrosioides *and *Kielmeyera neglecta *against *Artemia salina ***

		**LC**_50_**(μg/mL)**	
**Species**	**Extract**	**6 h**	**24 h**
*K. neglecta*	Hex	2699.8	1050.8
	DCM	- *	1379.2
	EtOAc	1324.8	780.8
	EtOH	2154.5	977.7
*C. ambrosioides*	Hex	- ^#^	- ^#^
	DCM	1371.2	359.9
	EtOAc	- *	1444.4
	EtOH	1059.0	356.6

Hex, hexane; DCM, dichloromethane; EtOAc, ethyl acetate; EtOH, ethanol. * No deaths enough for statistical analysis; ^#^ High mortality, invalidating the statistical test.

Antimicrobial activity was analyzed based on the results of MIC and IC 50% displayed in Table 2. It was possible to determine the minimal inhibitory concentration only for the extract from *C. ambrosioides* Hex (100 μg/mL) against *C. krusei*, which has clearly shown complete inhibition; when examining the reading for 50% CI, the concentration was 3.1 μg/mL. For some antifungals, the reading of MIC is based on 50% of growth inhibition [34]; then, the reading of IC 50% should be considered when screening plants with antimicrobial activity. An antimicrobial activity can be considered as positive when the action of the extract occurs at concentrations lower than 100 μg/mL [10]; hence, when considering the reading of IC 50%, five extracts are regarded as very important against at least one organism; on the other hand, when analyzing MIC, only *C. ambrosioides* Hex was considered significant. Despite the MIC and IC 50% at 100 μg/mL for *C. ambrosioides* Hex and DCM, respectively, the genuine inhibitory concentration is between that concentration read in the test and the one next lower tested concentration [33]; then, its true 50% MIC/IC is lower than 100 μg/mL. When analyzing the reading of IC 50%, extracts from *C. ambrosioides* have not shown antibacterial activity, but antifungal activity with Hex and DCM. In turn, *K. neglecta* showed antibacterial activity against Gram positive bacteria, as well as antifungal activity against non-*albicans Candida* species (Hex, EtOAc, EtOH). It is noteworthy that extracts from *K. neglecta* EtOAc and EtOH had the same IC 50% when tested with *E. faecalis* ATCC 51299 and *S. aureus* ATCC 43300 multidrug-resistant bacteria.

**Table 2:** **Antimicrobial activity of extracts of *K. neglecta *and *C. ambrosioides***

	**MIC/IC 50% (μg/mL)**							
**Microorganisms**	***K. neglecta***				***C. ambrosioides***			
	**Hex**	**DCM**	**EtOAc**	**EtOH**	**Hex**	**DCM**	**EtOAc**	**EtOH**
*E. faecalis* ATCC 29212	−/−	−/−	−/−	-/12.5	−/−	−/−	−/−	−/−
*E. faecalis* ATCC 51299	−/−	−/−	−/−	-/12.5	−/−	−/−	−/−	−/−
*E. coli* ATCC 25922	−/−	−/−	−/−	−/−	−/−	−/−	−/−	−/−
*E. coli* ATCC 35218	−/−	−/−	−/−	−/−	−/−	−/−	−/−	−/−
*K. pneumoniae* ATCC 700603	−/−	−/−	−/−	−/−	−/−	−/−	−/−	−/−
*P. aeruginosa* ATCC 27853	−/−	−/−	−/−	−/−	−/−	−/−	−/−	−/−
*S. aureus* ATCC 29213	-/12.5	−/−	-/12.5	-/12.5	−/−	−/−	−/−	−/−
*S. aureus* ATCC 43300	−/−	−/−	-/12.5	-/12.5	−/−	−/−	−/−	−/−
*C. albicans* ATCC 90028	−/−	−/−	−/−	−/−	−/−	-/300	−/−	−/−
*C. albicans* ATCC 10231	−/−	−/−	−/−	−/−	-/50	-/300	−/−	−/−
*C. parapsilosis* ATCC 90018	−/−	−/−	-/300	−/−	-/50	−/−	−/−	−/−
*C. parapsilosis* ATCC 22019	−/−	−/−	-/300	−/−	-/50	-/300	−/−	−/−
*C. krusei* ATCC 6258	-/500	−/−	-/200	-/200	100/3.1	-/100	−/−	−/−

*Hex*, hexane; *DCM,* dichloromethane; *EtOAc*, ethyl acetate; *EtOH*, ethanol. (−) No inhibition at the concentrations tested. *MIC,* minimum inhibitory concentration. IC 50%, inhibitory concentration of 50% growth.

As the extract from *C. ambrosioides* Hex was the only one with complete inhibition of visible growth, the evaluation of MFC was only performed for this extract. With a final concentration of 200 μg/mL, this extract was considered to exert a fungicidal effect against *C. krusei*. This yeast is regarded as intrinsically resistant to fluconazole and resistant to other antifungals such as itraconazole and flucytosine [41]; this is an interesting result because, as there is an activity targeted against *C. krusei*, the compounds present in this extract can be used in the formulation of drugs for an specific treatment of candidiasis caused by this species, which has increased over the last years [41,42]. The different concentrations of MIC (100 μg/mL) and MFC can be explained by the reading of visible growth, without automation, as suggested by the CLSI [34]; that may result in some variations in results.

The extracts that showed antimicrobial activity against the tested yeasts, *K.neglecta* (Hex, EtOAc and EtOH) and *C. ambrosioides* (Hex and DCM) were evaluated as for the action on the cell wall of *N. crassa*, but showed no inhibition halo at the tested concentration, either antifungal action related to cell wall inhibition. Despite no action has been detected on cell wall using this method, it is possible that antifungal action occurs through another mechanism.

**Figure 2:**
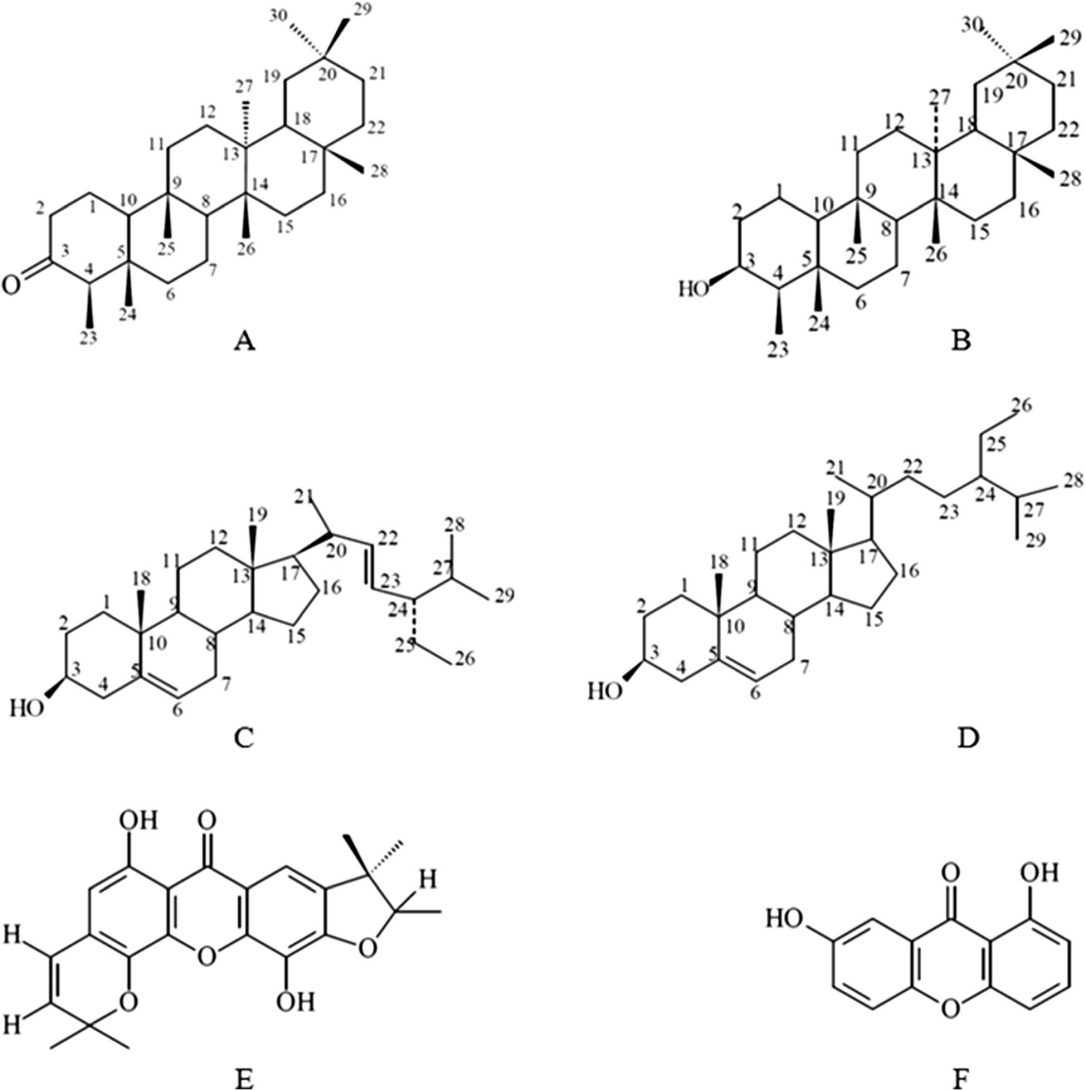
**Chemical structures identified from *K. neglecta*: friedelan-3-one (A), friedelan-3-β-ol (B), β-sitosterol (C), stigmasterol (D), 1,8-dihydroxy-6,6-dimethylpyrano(2,3;5,6)-4′,4′,5′-trimethyldihydrofuran(2′,3′;2,3)xanthone (E), 1,7-dihydroxyxanthone (F).** With permission of the author. (Oliveira, unpublished results).

A preliminary chemical study of extracts of roots and branches from *K. neglecta* resulted in the isolation of 24 substances which identification and determination of structures by spectrometric methods (UV, IR, ¹H NMR, ¹³C NMR, NOESY) and comparison with data from the literature were done. Isolated substances were the triterpenes friedelan-3-one (friedelin) (Figure 2A) and friedelan-3-β-ol (friedelinol) (Figure 2B); the steroids β-sitosterol (Figure 2C) and stigmasterol (Figure 2D); and the xanthones 1,8-dihydroxy-6,6-dimethylpyrano(2,3;5,6)-4′,4′,5′-trimethyldihydrofuran(2′,3′;2,3)xanthone (Figure 2E) and 1,7-dihydroxyxanthone (Figure 2F) [43]. The analysis of biological activities with the isolated compounds was not performed, but it might be possible that the xanthones present in *K. neglecta* are synergistically responsible for the antifungal activity presented in this study, as some xanthones isolated from *K. coriacea* have proven to have antifungal activity against *Cladosporium cucumerinum* and *C. albicans*, whereof some have shown a fungicidal effect against the latter [44]. Antimicrobial activity against *Candida* species can also be explained by the compound friedelin, which has been shown to have activity against *C. albicans, C. krusei* and *C. glabrata*[45]. In addition, friedelin has shown to have microbicidal activity against *E. faecalis*[45] and *S. aureus*[46], whereas friedelinol has shown to have microbicidal activity against *S. aureus*[46].

A preliminary qualitative chemical analysis of extracts of *C. ambrosioides* obtained in this study, conducted according to Matos [47], revealed the presence of phenolic compounds, tannins, flavonoids and steroids for the EtOH extract, phenolic compounds, tannins and flavonoids for the EtOAc extract and flavonoids and steroids for the DCM extract. Ascaridol has been the major antifungal compound identified in extracts of *C. ambrosioides*, with activity against *Aspergillus flavus*, *A. glaucus*, *A. ochraceous*, *A. niger*, *Fusarium semitectum*, *F. oxysporum*, *Colletotrichum musae* and *C. gloeosporioides*. [17,24], being probably this compound the responsible for the activity of *C. ambrosioides* against *Candida* species in ours study.

## Conclusion

This study has demonstrated that these two plants - *K.neglecta* and *C. ambrosioides* - used in the Brazilian folk medicine are a potential source of substances with biological activities. By showing bioactivity against *A. salina*, *K. neglecta* EtOAc and EtOH and *C. ambrosioides* Hex, DCM and EtOH may be considered extracts that potentially contain substances with cytotoxic activity against cancer cells. The IC 50% determination has been proved to be useful in the screening of extracts with antimicrobial activity and should be considered as an important tool in researches involving natural products. Extracts from *K. neglecta* EtOAc and EtOH showed activity against multidrug-resistant bacteria, which are of great concern in medicine nowadays; in turn, *C. ambrosioides* Hex and DCM showed specific activity against yeasts. These results showed here confirm once more the *K. neglecta* and *C. ambrosioides* extracts potential biological activities.

## Competing interests

The authors declare that they have no competing interests.

## Authors’ contributions

ZLS performed the extraction, the laboratory assays and drafted the manuscript. FFO participated in the design of the chemical study. AOC helped to draft the manuscript. LAMS performed the collection and identification of plant material. MHR and JSS conducted preliminary chemical analysis. JLA participated in the design and coordination of the study. All authors read and approved the final manuscript.
